# Interaction between neutrophil extracellular traps and cardiomyocytes contributes to atrial fibrillation progression

**DOI:** 10.1038/s41392-023-01497-2

**Published:** 2023-07-26

**Authors:** Li He, Ruiqi Liu, Honghua Yue, Xiaoxin Zhang, Xiaohui Pan, Yutao Sun, Jun Shi, Guonian Zhu, Chaoyi Qin, Yingqiang Guo

**Affiliations:** 1 Department of Cardiovascular Surgery and Cardiovascular Surgery Research Laboratory, West China Hospital, Sichuan University, Chengdu, 610041 Sichuan China; 2 Department of Plastic and Burn Surgery, West China Hospital, Sichuan University, Chengdu, China; 3 West China Center of Excellence for Pancreatitis, Institute of Integrated Traditional Chinese and Western Medicine, West China Hospital, Sichuan University, Chengdu, China; 4 Department of Endocrinology & Metabolism, West China Hospital, Sichuan University, Chengdu, China; 5 Department of Pulmonary and Critical Care Medicine and Institute of Respiratory Health, West China Hospital, Sichuan University, Chengdu, China

**Keywords:** Cardiology, Cardiovascular diseases, Innate immunity, Innate immune cells

## Abstract

Atrial fibrillation (AF) is a frequent arrhythmia associated with cardiovascular morbidity and mortality. Neutrophil extracellular traps (NETs) are DNA fragments with cytoplasm proteins released from neutrophils, which are involved in various cardiovascular diseases. To elucidate the role of NETs in AF, we investigated the effect of NETs on AF progression and the secretion of NETs in AF. Results showed that: NETs induced the autophagic apoptosis of cardiomyocytes, and NETs also led to mitochondrial injury by promoting mitochondrial depolarization and ROS production. Ongoing tachy-pacing led to the structural loss of cardiomyocytes and provided potent stimuli to induce NETs secretion from neutrophils. In the meanwhile, increased Ang II in AF facilitated NETs formation through the upregulation of AKT phosphorylation, while it could not directly initiate NETosis as the autophagy was not induced. In vivo, DNase I was administrated to abrogate NETs formation, and AF-related fibrosis was ameliorated as expected. Correspondingly, the duration of the induced AF was reduced. Our study addresses the formation mechanism of NETs in AF and demonstrates the lethal effects of NETs on cardiomyocytes through the induction of mitochondrial injury and autophagic cell death, which comprehensively describes the positive feedback comprised of NETs and stimuli secreted by cardiomyocytes that sustains the progression of AF and AF related fibrosis.

## Introduction

Atrial fibrillation (AF) is the most common arrhythmia characterized by rapid, irregular beating of the atria. In China, the prevalence of AF is 1.8 percent, which increases significantly in the elderly population.^1^ Epidemiological studies have shown the prevalence of AF increases significantly after 60 years of age, and about 45 percent of AF patients in China are over 65 years old.^2–4^ Cardiac dysfunction and stroke caused by thrombosis are the most common complications of AF and are detrimental, especially for stroke that may lead to disability and even death, increasing the social and economic burden.

Based on the latest publications, the main treatments for AF include medication, catheter ablation and surgical ablation.^5^ Clinical studies have shown the 5-year recurrence rate for catheter ablation and conventional radiofrequency are 71 and 40 percent, respectively.^6–8^ Even though inflammation and fibrosis essentially orchestrate the pathogenesis and persistence of AF, the mechanism is presently far from being elucidated.^9^ Therefore, the further clarification of specific mechanisms of AF and the development of effective methods to control it are critical.

The occurrence and progression of AF are closely related to the inflammatory reaction and immune cell activation. The main cell type involved in the innate immune response is neutrophils, whose main role was believed to eliminate pathogenic bacteria and injured cells in the acute inflammatory response.^10^ However, neutrophils have recently been demonstrated to secret neutrophil extracellular traps (NETs) to participate in the occurrence and development of multiple diseases. By now, chronic obstructive pulmonary disease, atherosclerosis, hepatitis, and many other sterile chronic diseases are associated with the formation of NETs, but whether NETs play a role in AF is still unclear.^11–15^

NETs are a network structure with DNA fragments as the skeleton. The DNA skeleton of NETs contains nuclear and mitochondrial DNA, with abundant neutrophil granules and cytoplasm proteins scaffolded.^16^ Previously we described that NETs sustain pathogenic fibrosis after acute myocardium infarction (AMI) and contribute to the formation of ventricular aneurysm through the activation of Smad and mitogen-activated protein kinase (MAPK) signaling pathways.^17^ As fibrosis is closely related to AF, the involvement of NETs in AF was investigated in this study. Since cardiac remodeling involves both the loss of tissue cells in situ and the proliferation of fibroblasts, we examined the lethal effects of NETs on cardiomyocytes. In addition, we also investigated whether NETosis could be initiated by stimuli secreted by cardiomyocytes in experimental in vitro model of AF.

## Results

### NETs are involved in atrial fibrillation

**Fig. 1:**
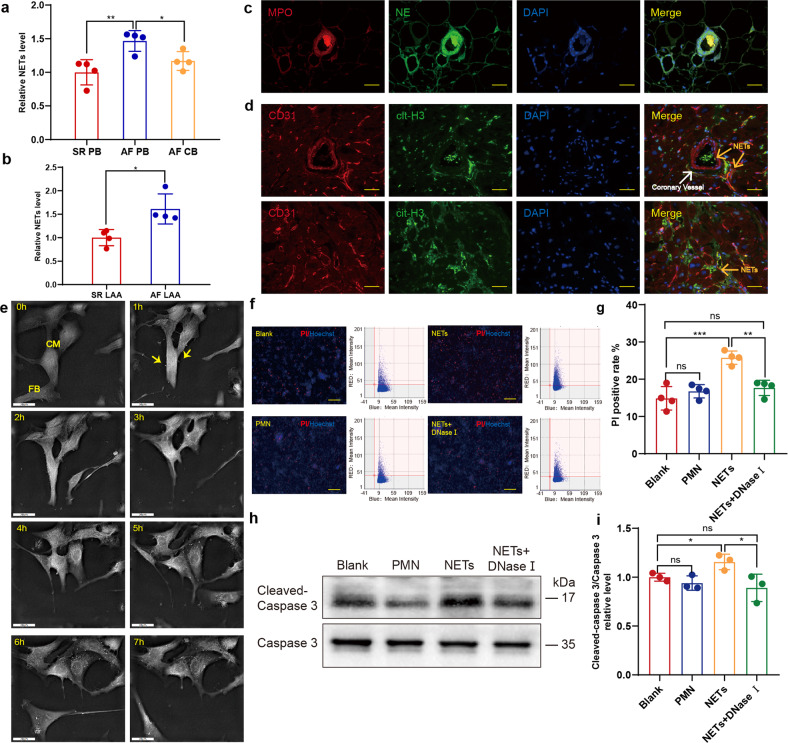
NETs are increased in AF patients and cytotoxic to cardiomyocytes. **a** Complex enzyme-linked immunosorbent assay (ELISA) experiment comparing NETs in the peripheral blood and coronary blood of patients with AF or SR (*n* = *4*). **b** Comparison of NETs concentration in left atrial appendage homogenates of patients with AF and SR (*n* = *4*). **c** NETs formed in the coronary vessels of AF patients. LAA section was stained for DNA (blue), NE (green) and MPO (red). Scale bar: 100 μm. MPO and NE double positive structure was defined as NETs. **d** NETs formed outside the coronary vessels of AF patients. LAA section was stained for DNA (blue), cit-H3 (green) and CD31 (red). Scale bar: 100 μm. Coronary vessels were labelled with CD31 and NETs were identified as cit-H3 positive. **e** Cardiomyocytes incubated with NETs underwent atrophy and perinuclear granule increase. Yellow arrows indicate NETs binding to the surface of cardiomyocytes. Scale bar: 20 μm. **f, g** Death rate of cardiomyocytes measured by Celigo analysis in the presence or absence of NETs and supernatants of PMN (*n* = *4*). Cells in 96well plate were stained for all DNA (blue) and nucleuses of dead cells (red). Scale bar: 150 μm. Hoechst single positive cell was defined as the living, while Hoechst and PI double-positive cell was defined as the dead. **h**, **i** Increased cleaved caspase3/caspase 3 ratio in cardiomyocytes incubated with NETs analyzed through WB (*n* = *3*). SR sinus rhythm, AF atrial fibrillation, PB peripheral blood, LAA left atrial appendage, CB coronary blood, NE neutrophil elastase, MPO myeloperoxidase, PMN polymorphonuclear leukocytes, PI propidium iodide. *P < 0.05, **P < 0.01, ***P < 0.001, ns (not significant). Data were presented as mean ± SD

To investigate the possible relation between NETs and AF, we first collected the peripheral blood of patients with AF or sinus rhythm (SR). A complex enzyme-linked immunosorbent assay (ELISA) with antimyeloperoxidase (MPO) capture antibody and anti-dsDNA detective antibody was used to determine the relative concentration of NETs as previously described.^18^ As expected, the NETs level in peripheral blood in patients with AF was significantly higher than that of patients with SR (Fig. 1a). The NETs concentration in the homogenates of left atrial appendage was also compared, and a similar trend was observed (Fig. 1b). The coronary blood was collected at the coronary sinus orifice of patients with AF, and the NETs concentration was futher compared to that in peripheral blood. Interestingly, the NETs level decreased after blood passed through the coronary circulation (Fig. 1a). Thus we hypothesized NETs may possibly congested in coronary vessels, which is similar to the scenario of myocardial infarction (MI). To verify this hypothesis, immunofluorescent staining was performed in the left atrial appendage section to identify and locate NETs, which indicated the formation of NETs in left atrial appendages and the coronary vessels of patients with AF (Fig. 1c). However unlike mainly concentrating in coronary vessels after MI, NETs in AF also scattered beyond the coronary vessels (Fig. 1d).

### NETs lead to cardiomoyocyte apoptosis by promoting autophagy

Previously, we reported that NETs alone activate Smad and MAPK signaling pathways, which further induce the differentiation from fibroblast to myofibroblast. Considering that the loss of cardiomyocytes essential to cardiac remodeling and NETs contain abundant cytotoxic proteases, we further investigated whether NETs are toxic to cardiomyocytes. With Nanolive 3D-holomotomographic microscopy, we continuously monitored cardiomyocytes incubated with NETs, and atrophy along with perinuclear granule increase was observed in cardiomyocytes (Supplementary Movie 1; Fig. 1e). As some primary cardiomyocytes might fail to adhere to the plate and undergo apoptosis, cell remnants were regularly found sticking to the normal ones. While in flow cytometry, the clinging remnants may lead to the misjudgments of attached cells. To identify dead cardiomyocytes more precisely, combined staining with propidium iodide (PI) and Hoechst stain was analyzed through the Celigo whole view analysis instead. As expected, the PI-positive rate was elevated in NETs incubated wells (Fig. 1f, g), which was further supported by the similar trend of cleaved caspase3/caspase 3 detected through WB (Fig. 1h, i). These results indicated the cytotoxic effects of NETs on cardiomyocytes.

**Fig. 2:**
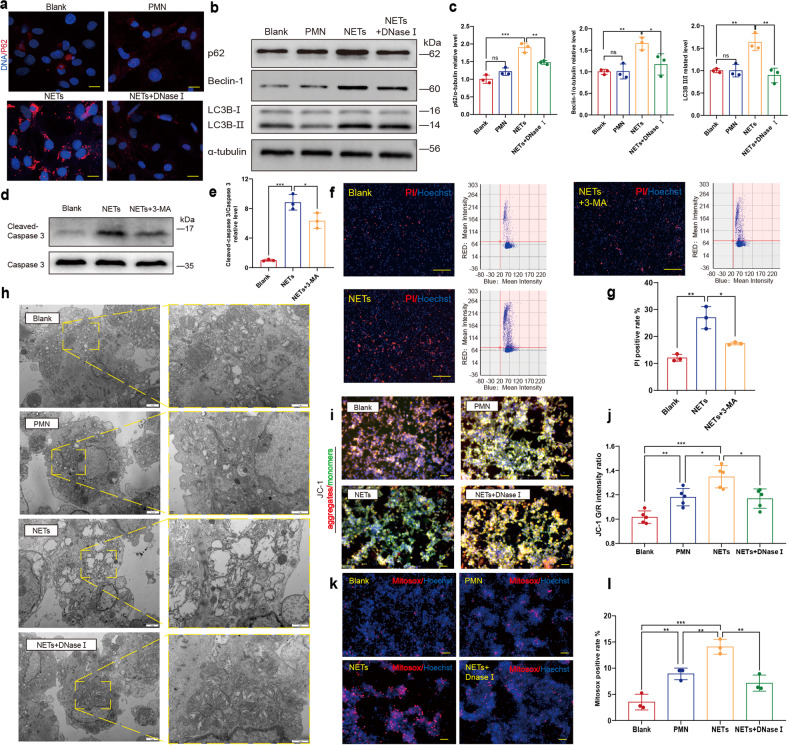
NETs induce the autophagic cell death and mitochondrial injury of cardiomyocytes through the upregulation of autophagy. **a** Increased p62 positive granules in cardiomyocytes incubated with NETs demonstrated by IF staining microscopy. Cells in 24-well plate on coverslips were stained for DNA (blue) and p62 (red). p62 positive granules were imaged with a confocal laser-scanning microscope. Scale bar: 10 μm. **b**, **c** In vitro p62, Beclin-1 and LC3B II/I were all upregulated in cardiomyocytes incubated with NETs (*n* = *3*). **d**, **e** In vitro 3-MA partly compromised the upregulation of cleaved- caspase 3 in cardiomyocytes induced by NETs (*n* = *3*). **f**, **g** Death rate of cardiomyocytes measured by Celigo analysis in the presence or absence of NETs and NETs+3MA (*n* = *3*). Cells in 96 well plate were stained for all DNA (blue) and nucleuses of dead cells (red). Scale bar: 100 μm. Hoechst single-positive cell was defined as the living, while Hoechst and PI double-positive cell was defined as the dead. **h** In vitro mitochondrial morphology damage was induced by NETs in cardiomyocytes, including swelling and reduction of cristae. Mitochondria were imaged with transmission electron microscope. Scale bar: 1 μm or 2 μm. **i**, **j** In vitro mitochondrial membrane potential was decreased in cardiomyocytes incubated with NETs (*n* = *5*). The mitochondrial membrane potential of cardiomyocytes in 6-well plate in the presence or absence of NETs, degraded NETs with DNase I and supernatants of PMN using JC-1 and Hoechst was imaged by fluorescence microscope. Scale bar: 50 μm. The mitochondrial membrane potential of cardiomyocytes in 96-well plate was measured by fluorescence microplate reader. **k**, **l** In vitro excessive mtROS was produced in cardiomyocytes incubated with NETs (*n* = *3*). Cells in 6-well and 96-well plate were stained for DNA (blue) and mtROS (red). mtROS of cardiomyocytes in 6-well plate in the presence or absence of NETs, degraded NETs with DNase I and supernatants of PMN using mitoSOX and Hoechst was imaged by Fluorescence microscope. Scale bar: 50 μm. Cardiomyocytes in 96well plate of increased mtROS production was compared by Celigo analysis. PMN polymorphonuclear leukocytes, mtROS mitochondrial reactive oxygen species. PI propidium iodide. *P < 0.05, **P < 0.01, ***P < 0.001, ns (not significant). Data were presented as mean ± SD

The perinuclear granules in cardiomyocytes increased significantly when incubated with NETs. As increased lysosomes will also relocate to the perinuclear region during autophagy, we further investigated whether autophagy was possibly involved in the NETs-induced cell death.^19^ Immunofluorescent staining of p62 demonstrated upregulation of autophagy after incubation with NETs (Fig. 2a). Accordingly, protein levels of p62 and Beclin-1 also increased (Fig. 2b, c). To further investigate whether NETs induce the autophagic cell death of cardiomyocytes, 3-methyladenine (3-MA) was used to block autophagy. As expected, 3-MA partly compromised the increased cleaved caspase3 (Fig. 2d, e). This protective effect of 3-MA against NETs-induced apoptosis of cardiomyocytes was also verified through PI staining and Celigo analysis (Fig. 2f, g).

### NETs lead to mitochondrial damage by promoting mitochondrial depolarization and reactive oxygen species production

As mitochondria are essential for the supply of energy for cardiomyocyte contraction whereas dysfunctional mitochondria with excessive reactive oxygen species (ROS) production can be death-promoting, the effects of NETs on cardiomyocyte mitochondria were analyzed.^20^ Swollen mitochondria in cardiac myocytes significantly increased after incubation with NETs (Fig. 2h). To further elucidate the detrimental effects of NETs on mitochondria, JC-1 and MitoSOX were used to compare the mitochondrial membrane potential and mitochondrial ROS (mtROS) production, respectively. As expected, NETs led to depolarization of the mitochondrial membrane potential (Fig. 2i, j). Mitochondrial ROS production in cardiac myocytes also increased after the incubation with NETs (Fig. 2k, l). In addition, we used CCK8 to verify the cytotoxic effect of NETs on cardiomyocytes, which was significantly different from the result of the PI staining that showed an opposite trend (Supplementary Fig. 1). As CCK-8 mainly detects dehydrogenation and NAD+ level in mitochondria, the increase in optical density (OD) after NETs incubation might be the result of the increased mitochondrial ROS production. And the deleterious effect of NETs on mitochondrial function was further investigated with WB analysis, demonstrating the increase of Bax and PINK1 and the decrease of Bcl-2 in cardiomyocytes incubated with NETs (Supplementary Fig. 2). However, unlike autophagy inhibition with 3-MA that protects cardiomyocytes from apoptosis, we found stabilizing mitochondria with mito-TEMPO led to further upregulation of cardiomoyctes apoptosis (Supplementary Fig. 3).

### Ang II alone cannot directly initiate NETosis

**Fig. 3:**
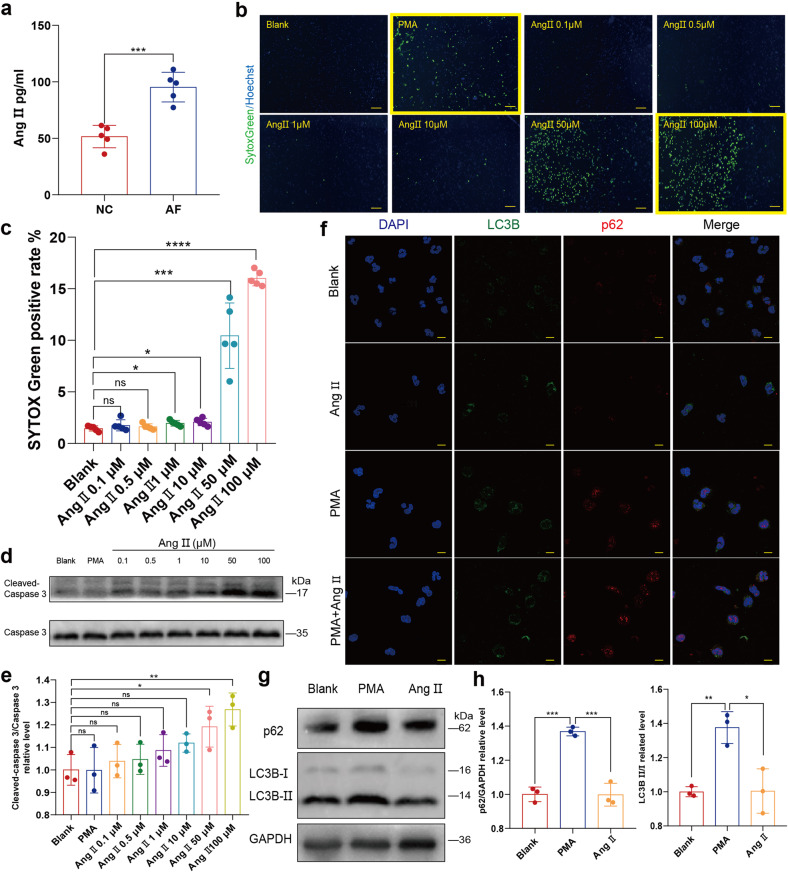
Ang II could not initiate NETosis. **a** ELISA experiment comparing Ang II in peripheral blood of patients with AF and normal control (*n* = *5*). **b**, **c** Neutrophils of cfDNA measured by Celigo analysis in the presence or absence of PMA and Ang II of different concentration (0.1 μM, 0.5 μM, 1 μM, 10 μM, 50 μM or 100 μM) (*n* = *5*). Neutrophils deprived from peripheral blood of the donator in 96-well plate was stained for all DNA (blue) and cfDNA (green). Scale bar: 100 μm. Hoechst single positive cell was defined as the living, while Hoechst and SYTOX Green double positive cell was defined as the neutrophil with cfDNA. Representatives with yellow frame demonstrate that despite the increase of SYTOX Green positive rate induced by Ang II, no linear or reticular DNA structure was identified. **d**, **e** Apoptosis analysis of Neutrophils incubated with PMA or Ang II of different concentration (0.1 μM, 0.5 μM, 1 μM, 10 μM, 50 μM or 100 μM) by comparing cleaved caspase 3/ caspase 3 ratio determined through WB(*n* = *3*). **f** In vitro autophagy was not upregulated in neutrophils incubated with Ang II alone. Neutrophils in 24-well plate on coverslips were stained for DNA (blue), LC3B (green) and p62 (red). p62 or LC3B positive subcellular structures were imaged with a confocal laser-scanning microscope. Scale bar: 10 μm. **g**, **h** Analysis of the effect of Ang II on autophagy in neutrophils by comparing p62 and LC3B II/I ratio determined through WB (*n* = *3*). Ang II angiotensin II, PMA Phorbol 12-myristate 13-acetate. *P˂0.05, **P < 0.01, ***P < 0.001, ****P < 0.0001, ns (not significant). Data were presented as mean ± SD

Inflammation can promote the progression of AF, and in turn, AF can promote inflammation. However, AF-related inflammation response is less strong compared to that of MI, in which abundant proinflammatory cytokines and oxidants induce NETosis. Apart from the inflammation-related stimuli, other molecules are ubiquitously made of the capability to induce NETosis. Akt is essential for NOX-dependent NETosis and phosphorylation of Akt is upregulated during NETosis.^21^ Previous studies demonstrated that elevated Ang II in AF induces cardiac remodeling, and phosphorylation of Akt is upregulated by Ang II type 1 (AT1) /receptor signaling pathway activation.^22^ Similarly to previous reports, we found that Ang II increased in the peripheral blood of patients with AF (Fig. 3a). Chrysanthopoulou et al. reported that 0.1 nM Ang II significantly induces NETosis in a ROS/autophagy-dependent manner.^23^ However, our results suggested that Ang II induces apoptosis rather than NETosis. We first compared the NETs secretion with SYTOX Green staining and Celigo whole view analysis, which demonstrated increased cell-free DNA (cfDNA) when neutrophils incubated with Ang II (Fig. 3b, c). However, the typical NETs structure was absent in the corresponding Celigo IF pictures (Fig. 3b).

Considering that the nucleus of dead cells can also be stained with SYTOX Green, we further determined the protein level of cleaved caspase 3 and found Ang II-induced apoptosis of neutrophils in a dose-dependent manner (Fig. 3d, e). Chrysanthopoulou et al. described that only a low Ang II concentration of 0.1 nM significantly increases the formation of NETs. However, when we used Celigo to compare SYTOX Green stained free DNA secreted from human neutrophils incubated with or without 0.1 nM Ang II, no significant difference was observed (Supplementary Fig. 4). In addition, we found Ang II did not upregulate the autophagy of neutrophils either (Fig. 3f-h).

**Fig. 4:**
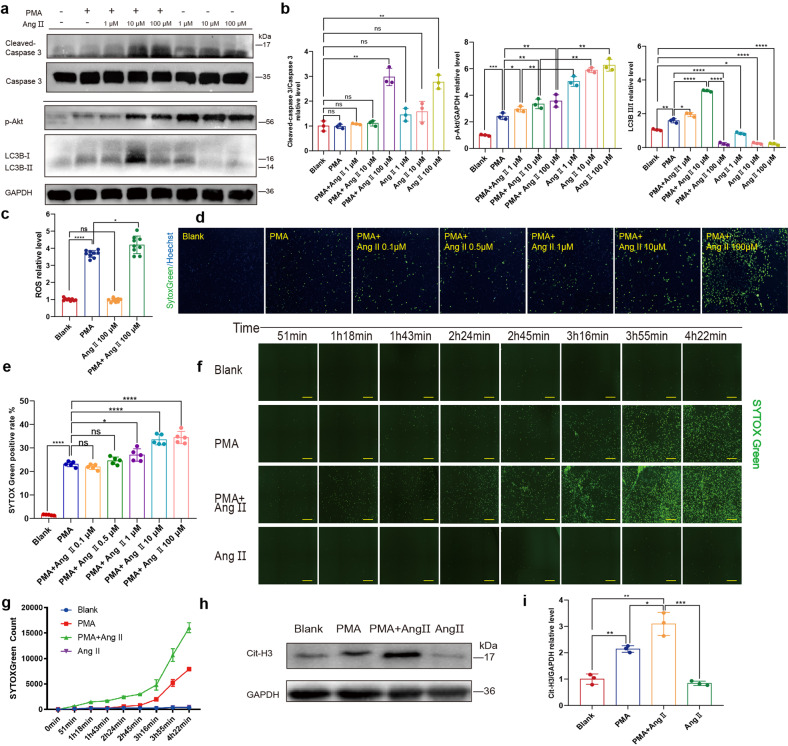
Ang II promotes NETosis and NETs secretion induced by PMA. **a**, **b** Apoptosis and autophagy analysis of human PMNs treated with PMA, Ang II of different concentrations (1 μM, 10 μM and 100 μM) or PMA+ Ang II. Analysis of p-Akt and LC3B II/I ratio (NETosis-related signaling) of PMNs treated with PMA, Ang II of different concentrations (1 μM, 10 μM and 100 μM) or PMA+ Ang II of different concentrations (1 μM, 10 μM and 100 μM) through WB (*n* = *3*). **c** In vitro Ang II further upregulated PMA-induced ROS production of PMNs (*n* = *9*). Suspended neutrophils were incubated with PMA, Ang II or PMA+ Ang II in 1.5 ml Eppendorf tubes gently shaking, and added into 96-well plate for subsequent determination of ROS with DCHF-DA in fluorescence microplate reader. **d**, **e** Analysis of concentration-dependent effect of Ang II on NETs secretion induced by PMA determined by Celigo (*n* = *5*). Rat neutrophils in 96-well plate treated with PMA, Ang II or PMA+ Ang II of different concentrations (0.1 μM, 0.5 μM, 1 μM, 10 μM and 100 μM) were stained for all DNA (blue) and cfDNA (green). Scale bar: 150 μm. Hoechst single positive cell was defined as the living, while Hoechst and SYTOX Green double positive cell with linear DNA structure formation was defined as cells undergoing NETosis. **f**, **g** Analysis of Time-dependent effect of 10 μM Ang II on NETs secretion induced by PMA determined by Celigo (*n* = *4*). Scale bar: 1 mm. **h**, **i** Analysis of cit-H3 in neutrophils in the presence or absence of PMA, Ang II and PMA+ Ang II through WB (*n* = *3*). Cit-H3, citrullinated histone 3. *P < 0.05, **P < 0.01, ***P < 0.001, ****P < 0.0001, ns (not significant). Data were presented as mean ± SD

Besides the increased phosphorylation of Akt, the activation of autophagy is also essential to NETosis. However, this phenomenon seems to be controversial as mTOR can be activated by phosphorylated Akt (p-Akt) and subsequently ameliorate autophagy.^24^ Thus, the potent stimuli for NETosis should be capable of activating both Akt phosphorylation and autophagy. Despite the upregulation of Akt phosphorylation, we found that Ang II inhibited autophagy rather than induce it (Fig. 4a, b).

### Ang II promotes PMA-induce NETosis

Phorbol myristate acetate (PMA), a common phorbol ester, strongly induces the activation of neutrophils. Since Ang II could not directly initiate NETosis, we then investigated the effect of Ang II on PMA-induced NETosis. Unlike Ang II immediately induce apoptosis of neutrophils, increased cleaved caspase 3 was not observed in rat neutrophils incubated with both PMA and Ang II (with a concentration less than 10 μM) (Fig. 4a, b). The phosphorylation of Akt was further upregulated when co-incubated with PMA and Ang II, however, the increase of phosphorylated Akt was not that significant when compared to the incubation with Ang II alone. Regarding autophagy, we found it was upregulated when neutrophils were co-incubated with both 10 nM PMA and 10 μM Ang II, however, a higher Ang II concentration (100 μM) seemed to reverse the pro-autophagy effect of PMA and Ang II alone abated LC3BII/I ratio in a dose-dependent manner (Fig. 4a, b). In addition, co-incubation led to higher ROS production in neutrophils (Fig. 4c). It seems that Ang II may play a pro-NETosis role when potent stimuli like PMA overwhelm its anti-autophagy effect. Therefore, we hypothesized Ang II may indirectly synergize stimuli like PMA to promote NETosis rather than directly initiate this specialized cell death.

Isolated neutrophils from the peripheral blood of donors were then incubated with PMA or PMA+ Ang II. As 10 μM Ang II did not induce significant apoptosis, this concentration was mostly employed in the subsequent experiments. Co-incubation with PMA and Ang II significantly increased SYTOX Green positive rate, which was more significant than that produced by Ang II or PMA alone (Fig. 4d, e). NETs secretion was further investigated with IF staining. After stimulation of neutrophils with PMA for 30 min, the decondensation of the nucleus was proceeding, which was not observed in neutrophils with or without the presence of Ang II only. Besides the decondensed nucleus, the process of NETosis was advanced when neutrophils were co-stimulated by PMA and Ang II, as NETs are commonly secreted (Supplementary Fig. 5). Similarly, when the concentration of angiotensin reached 100 μM, the NETs structures were also absent, and numerous lobulated nuclei (still condensed) were stained. Moreover, adding up the average SYTOX Green counts of PMA only and 10 μM Ang II only was far inferior to the SYTOX Green count of co-incubation, indicating the role of Ang II as a promoting factor (Fig. 4f, g). A similar trend was observed in histone 3 citrullination (Fig. 4h, i). Overall, Ang II could facilitate NETosis rather than directly initiate it.

### Supernatants of tachy-paced neonatal rat cardiomyocytes could induce NETosis as increased mitochondrial DNA and high mobility group box 1 secretion

**Fig. 5:**
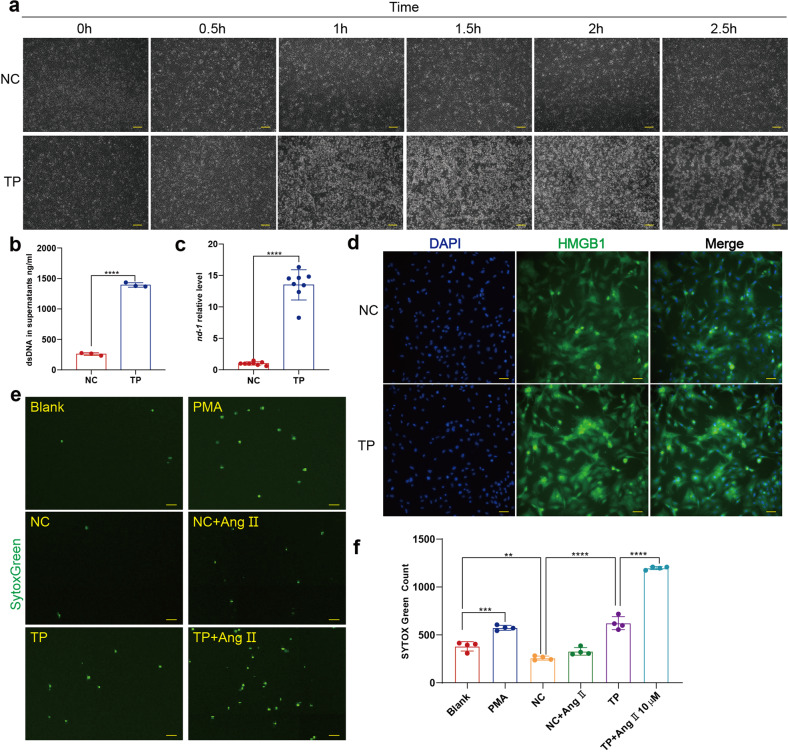
Supernatants of tachy-paced cardiomyocytes could induce NETosis. **a** Neonatal rat cardiomyocyte could not survive from long period of tachy-pacing. Cardiomyocytes in 6-well plate paced by C-Pace EP Culture Stimulator of 1 Hz (NC) or 6 Hz (TP) were observed and imaged by microscopy every 30 minutes. Scale bar: 200 μm. **b** Analysis of dsDNA with PicoGreen in the supernatants of tachy-paced cardiomyocytes and normal control (*n* = *3*). cfDNA in supernatants of tachy-paced cardiomyocytes and normal control were measured with PicoGreen incubated in 96-well plate by fluorescence microplate reader. **c** Analysis of mtDNA in the supernatants of tachy-paced cardiomyocytes and normal control (*n* = *8*). **d** HMGB1 was translocated from nucleus to cytoplasm in tachy-paced cardiomyocytes. Cardiomyocytes in 6-well plate were stained for DNA (blue) and HMGB1 (green). Scale bar: 20 μm. **e**, **f** Rat neutrophils undergoing NETosis measured by Celigo analysis in the presence or absence of PMA, Ang II and supernatants of cardiomyocytes paced with frequency of 1 Hz or 6 Hz (*n* = *4*). Rat neutrophils in 96-well plate were stained for all DNA (blue) and cfDNA (green). Hoechst single positive cell was defined as the living, while Hoechst and SYTOX Green double positive cell with decondensed nucleus was defined as cell undergoing NETosis. Scale bar: 500 μm. TP, tachy-pacing, HMGB1 high mobility group box 1. **P < 0.01, ***P < 0.001, ****P < 0.0001. Data were presented as mean ± SD

As elevated Ang II alone did not induce NETosis, other possible sources of stimuli must exist to induce NETosis in AF. Mallavia et al. reported that NETs formation could be triggered by mitochondrial DNA (mtDNA), and Wiersma et al. demonstrated that cardiomyocytes undergo mitochondrial dysfunction after tachy-pacing.^25,26^ Therefore, we hypothesized that tachy-paced cardiomyocytes may release mtDNA to induce NETosis. To prove this hypothesis, we conducted tachy-pacing (6 Hz, 40 V, 10 ms pulses) as experimental AF or normal pacing as negative control (1HZ, 40 V, 10 ms pulses) on neonatal rat cardiomyocytes as described previously, and the supernatants were collected (Supplementary Movie 2).^27^ The pacing process was continuously monitored under microscopy and we found that the cardiomyocytes did not survive from continuous tachy-pacing as structural damage was observed after 1 h of the electrical stimulation (Fig. 5a). We first compared the cfDNA and mtDNA levels between the two groups, and found they both increased in the supernatants after tachy-pacing of 3 hours (Fig. 5b, c). In addition, mtDNA as one part of cfDNA increased more dramatically than cfDNA. Besides the elevated mtDNA, we found translocation of high mobility group box 1 (HMGB1) from the nucleus to the cytoplasm could also be induced by tachy-pacing, which is of the potent capability to trigger NETosis (Fig. 5d).^28^

In the next, isolated rat neutrophils were incubated with PMA, supernatants of normally paced cardiomyocytes, supernatants of tachy-paced cardiomyocytes, and supernatants of tachy-paced cardiomyocytes + Ang II. Rat neutrophils are prone to secrete cloudy rather than the spiky NETs, and are weak in cross-linking to form a greater web-like structure, which is difficult to identify and quantify through IF microscopy.^29^ Thus, Celigo whole view analysis was used to analyze NETs secretion through SYTOX Green staining of extracellular DNA. As expected, the SYTOX Green count increased after incubation with supernatants of tachy-paced cardiomyocytes and more significantly with additional administration of Ang II, which was similar compared to that of PMA (Fig. 5e, f). And in several cases, the supernatants of tachy-paced cardiomyocytes were even more potent than 500 nM PMA in inducing NETosis (Supplementary Fig. 6). Notably, supernatants of normal-paced cardiomyocytes reduced NETosis, which indicated that normal cardiomyocytes may secrete some kind of cytokines to protect neutrophils from NETosis.

### DNase I intravenous injection downregulates NETs formation, ameliorates AF-related fibrosis and decreases the AF duration

**Fig. 6:**
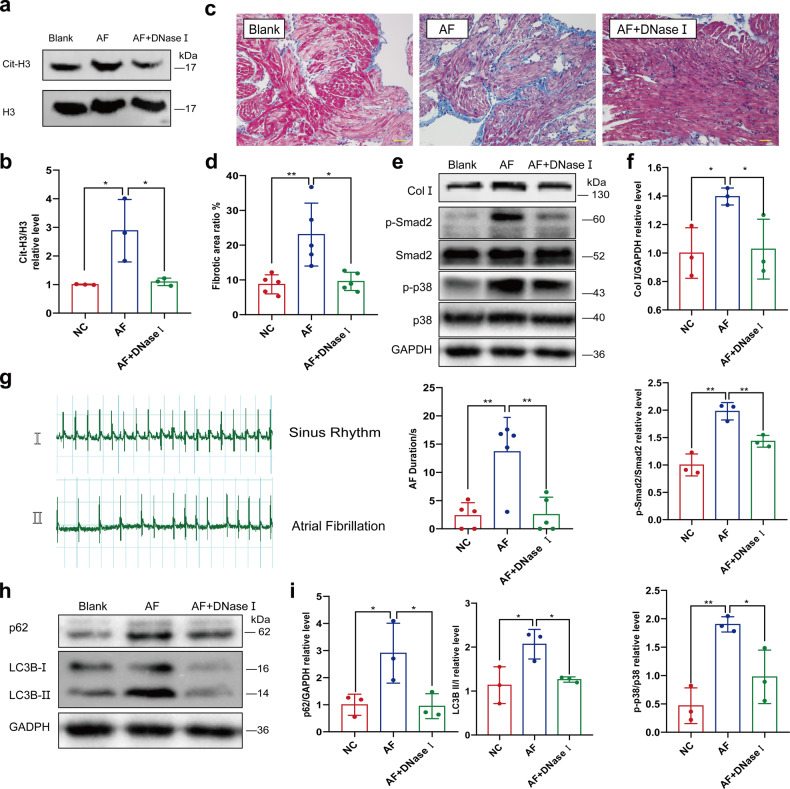
DNase I intravenous injection ameliorates AF-induced atrial fibrosis. **a**, **b** In vivo DNase I abrogated AF-related upregulation of cit-H3 (*n* = *3*). Cit-H3 in left atrium of rats with or without pharmacal induction of AF and DNase I injection was compared through WB. **c**, **d** In vivo DNase I ameliorated AF-related fibrosis (*n* = *5*). Fibrotic area of rat left atrium was analyzed with Masson’s Trichrome staining. Blue area was defined as the fibrotic, red area was defined as normal myocardium. Scale bar: 50 μm. **e**, **f** Analysis of Collagen I, p-p38 and p-smad2 in left atrium of rat with or without pharmacal induction of AF and DNase I injection determined through WB (*n* = *3*). **g** In vivo DNase I ameliorate the prolonged AF duration by continual pharmacal induction of AF (*n* = *5*). AF induced by Ach-CaCl_2_ in rats with or without continual pharmacal induction of AF and DNase I injection was recorded by ECG and AF duration was documented. **h**, **i** In vivo upregulated autophagy by pharmacal induction of AF could be partly compromised by DNase I injection (*n* = *3*). Analysis of p62 and LC3B II/I ratio in the left atrium of rat with or without pharmacal induction of AF and DNase I injection determined through WB. ECG, electrocardiograph. *P < 0.05, **P < 0.01. Data were presented as mean ± SD

NETs are a network structure with dsDNA as its skeleton, which can be degraded by DNase I. Therefore, we investigated whether DNase I had a protective effect and could decrease AF-related fibrosis and AF duration. AF was induced in rats through intravenous injection of a mixed solution of calcium chloride and acetylcholine, and the electrocardiogram (ECG) was recorded to ensure a successful induction. The injection was administrated every other day for two fortnights, along with additional injection of DNase I solution or the same volume of normal saline. The formation of rat NETs was compared with citrullinated Histone 3 (cit-H3) in the left atrium (LA) determined through western blot (Fig. 6a, b). Masson trichrome staining demonstrated that induced AF led to increased collagen deposition in LA, which was relieved by additional administration of DNase I (Fig. 6c, d). A similar trend was observed in the protein levels of collagen I (Col I), phosphorylated-Smad2 (p-smad2) and phosphorylated p38 (p-p38) (Fig. 6e, f). In addition, DNase I was effective in ameliorating the success rate and duration of induced AF (Fig. 6g). Since NETs induced autophagy in cardiomyocytes, we also determined autophagy-related proteins such as p62, Beclin-1 and LC3B in LA. As expected, DNase I blocked the AF-related upregulation of autophagy (Fig. 6h, i).

## Discussion

Recently, NETs have been widely investigated as a pathogenic factor that contributes to different diseases (including autoimmune disorders, cardiovascular diseases, and pulmonary diseases) through multiple mechanisms. Of all these potential mechanisms, innate immune response and cardiac fibrosis after MI have been investigated most extensively. A comparison of the ST-elevation myocardial infarction (STEMI) patients with low-level and high-level NETs uncovered that NETs would be positively correlated with excessive systemic inflammation in STEMI patients, while negatively associated with the matured atheroma.^17^ Furthermore, Mangold et al. have demonstrated that NETs are important determinants of microvascular occlusion (MVO) formation in STEMI patients.^30^ Colchicine has been found to be efficient in improving cardiac function through the reduction of cardiac remodeling after MI as its inhibiting effect on NETosis.^31^ In addition, we also found that NETs activate myofibroblast differentiation and participate in the formation of ventricular aneurysm after MI.^32^ However, Wei et al. indicate that the absence of EDIL3 leads to increased NETs formation, which mediates the differentiation of macrophages and benefits the longstanding fibrosis after MI.^33^ This is probably related to the action of S100A9: long-term S100A9 blockade negatively impacts cardiac recovery^34^ and counterbalances the beneficial effects of short-term therapy.^35^ And in this study, we described the elevated NETs concentration in both the peripheral blood and LAA of patients with atrial fibrillation when compared to patients with SR. Possibly due to the combined pathological conditions, there was no significant difference of infiltrated neutrophils identified between patients with or without AF (Supplementary Fig. 7). In addition, it is a common phenomenon in AF that lots of neutrophils could infiltrate into the myocardium from the pericardium.

Besides fibroblast activation, the loss of cardiomyocytes is also essential to cardiac remodeling. Mitochondrial dysfunction has been found to contribute to AF initiation and progression, which is caused by mitochondrial Ca^2+^ overload and excessive mitochondrial ROS production.^36^ In this study, we demonstrated that NETs could induce the mitochondrial injury of cardiomyocytes. As mitochondrial injury and cardiac apoptosis are not evident in AF rats, we instead performed the experimental model of AF in vitro (tachy-pacing with 6 Hz as AF compared to normal pacing with 1 Hz as the control), which is well established.^26,27^ In the meanwhile, structural damage was observed in tachy-paced cardiomyocytes, and the supernatants with increased mtDNA induced the secretion of NETs. Once AF occurred, cardiomyocytes with mitochondrial dysfunction would secret mtDNA along with other stimuli to activate NETosis, and NETs in turn will further deteriorate the mitochondrial injury, which as a whole forms the positive feedback loop to sustain NETs formation and mitochondrial injury. As NETs could promote the myofibroblast differentiation and fibrosis, AF-related fibrosis will also be sustained thereafter. However, the damage caused by AF on the myocardium seems to be less severe in vivo and other protective mechanisms may exist to reduce the mitochondrial injury and cardiac apoptosis.

In this research, we also demonstrated the upregulation of autophagy in cardiomyocytes after NETs incubation. Yuan et al. demonstrated autophagy-related gene7 (ATG7) is significantly upregulated in AF patients, and ATG7 knockdown restores the shortened atrial effective refractory period and alleviates the AF vulnerability.^37^ In our study, autophagy contributed to the NETs-induced death of cardiomyocytes. In rats, induction of AF also resulted in autophagy, whereas Dnase I administration compromised the upregulation of autophagy and decreased the duration of induced AF. We also analyzed the possible toll-like receptors (TLRs) activated by NETs, of which TLR4 was significantly upregulated (Supplementary Fig. 8). The substantial effect of the TLRs signaling pathway activation on cardiomyocytes remains controversial.^38–42^ We primarily hypothesized TLR4 signaling was activated by NETs and led to the apoptosis of cardiomyocytes. However, the knockdown of TLR4 resulted in increased NETs-mediated cardiomyocyte death, which demonstrated the protective role of TLR4 in the process (Supplementary Fig. 9). This assumption about the protective effect of TLR4 was also supported by the work of Lee et al. who demonstrated autophagy is suppressed by TLR4 activation through inhibition of FOXO3.^43^ In addition, autophagy is essential to NETosis and the profibrogenic effect of NETs on fibroblast as well.^44,45^ Recently, we found autophagy contributes to pathogenic fibrosis after myocardium infarction (MI), which is induced by NETs (data not shown). In fibroblast, sting signaling pathway is activated by internalized NETs and further increases the phosphorylation of TANK-binding kinase 1 (TBK-1) and interferon regulatory factor 3 (IRF-3), which is protective as it blocks the phosphorylation of smad3.^46^ Sting-mediated autophagy degrades phosphorylated TBK-1 (p-TBK-1) and abrogates the protective effect of the sting signaling pathway.^47^ When NETs are abundantly accumulating, the inhibition of autophagy may be protective, which restrains fibroblast differentiation and reduces myocardial injury. Therefore, pharmacological intervention against autophagy may be protective against fibrosis in the initiation and persistence stage of AF.

The pro-fibrosis effect of NETs in cardiovascular disease may not be limited to its direct effects on cardiomyocytes or fibroblasts. On NETs, many active scaffolded proteases may transform the proinflammatory cytokines from the precursor to the activated form. We identified the profibrogenic role of IL-36γ after MI, which activated the MAPK signaling pathway through IL-36R activation. The initial level of the IL-36γ in normal mice reached 2 μg per 0.1 g myocardium; however, despite the significant upregulation of the IL-36R signaling pathway, IL-36γ in myocardium did not increased significantly after MI (data not shown). The possible regulation mechanism of IL-36γ after MI may be that NETs with cathepsin G and elastase are secreted and transform pro-IL-36γ into the activated form. Besides cytokine activation, NETs promote thrombo-inflammation and lead to vascular occlusions.^18,48^

In LA of patients with AF, we identified micro-thrombi with NETs in the outer layer. During thrombosis neutrophils are recruited and activated to secrete NETs by the von Willebrand factor (VWF) and P-selectin deprived from the endothelium, which promotes thrombosis by providing scaffold and recruiting of XIIa factor, platelets, and red blood cells.^49–51^ The formation of micro-thrombus further facilitates NETs formation and result in the ischemia of the myocardium and the mitochondrial dysfunction of cardiomyocytes.^48^ In addition, we identified NETs in the mural thrombi obtained from LA of patients with AF (Supplementary Fig. 10). However, NETs are mainly present in the middle of the mural thrombus rather than the outer layer. For the close relationship between NETs and thrombogenesis, the promising intervention for AF aims at NETs may also be established on the modulation of forming NETs to inhibit thrombogenesis.

## Materials and methods

### Rats

Specific-pathogen-free (SPF) Sprague-Dawley (SD) rats, weighing 250 g, were purchased from Dashuo Biomedical Technique Company (Chengdu, Sichuan, China). Rats were housed under SPF conditions and grown in an environment of appropriate temperature and humidity, with sufficient water and food. The animal experiments were performed in compliance with the guidelines of the Ethics Committee of West China Hospital of Sichuan University under a project license (No. 20211404 A),

### Patient sample collection

The study was approved by the Ethics Committee of West China Hospital of Sichuan University and followed the principles of the Helsinki Declaration. All samples were obtained after informed consent. Peripheral blood was obtained before surgery, while coronary blood was obtained at the coronary sinus orifice in the short time window before cardiopulmonary bypass. Sera obtained from blood samples through centrifugation were stored at −80 °C. LA tissue was divided into 0.1 g portions and homogenized with 1 mL PBS, centrifuged at 3000 rpm for 30 min, and stored at −80 °C.

### NETs quantification

Quantification of NETs in sera and homogenates was performed using a complex ELISA combining anti-MPO (ab134132, Abcam, USA) as capture antibody and biotin-conjugated anti-dsDNA (catalog no. 11544675001, component 2; Cell Death ELISAPLUS, Roche, USA) as detective antibody.^18^

### Immunostaining and microscopy

LA was embedded in paraffin and used for immunohistochemistry or immunofluorescence (IF) analyses. Masson’s trichrome was used to compare the collagen volume. For IF staining of heart sections, primary antibodies anti-MPO (ab90810 or ab134132, Abcam, UK), neutrophil elastase (NE, ab131260, Abcam, UK) and cit-H3 (ab5103, Abcam) antibodies were used to identify NETs in LA. Cells were fixed with 4% paraformaldehyde for 15 min and NETs were fixed for 30 min to 1 h. Followed by permeabilization with 0.5% Triton-X-100 (BioFroxx, Germany) for 10 min, and sealing with the corresponding serum (Solarbio, China). Besides using primary antibodies to identify NETs, anti-LC3B (2775 S, CST, USA) and anti-p62 (ab56416, Abcam) antibodies were incorporated for the analysis of autophagy. The primary antibodies were incubated overnight at 4 °C, while the secondary antibodies were incubated at room temperature for 1 h in a dark room. DAPI (10 μg/mL) was used to stain nuclei.

### Neonatal rat cardiomyocytes isolation and culture

Cardiomyocytes were isolated from the hearts of neonatal SD rats. Hearts were cut into patches of 1 mm^3^, repeatedly digested in 5 mL PBS containing 1% trypsin (15090046, Gibco, USA) and collagenase type II (LS004176, Worthington, USA) at 37 °C for 10 min, and stopped by transferring the supernatants to an equal volume of Dulbecco’s modified Eagle’s medium (DMEM, C11995500BT, Gibco, USA) with 10% fetal bovine serum (FBS; 10099141 C, Gibco, Australia). The cell suspension was centrifuged at 1000 rpm for 10 min and cells were resuspended in DMEM/low glucose supplemented with Glutamax (10567014, Gibco, USA), 10% FBS and 1% penicillin-streptomycin (PS; SV30010, Hyclone, USA), from which the fibroblasts were further removed through differential adherence for 30 min. The supernatants were then transferred to a six-well plate and cultured at 37 °C and 5% CO_2_ for 24 hours, after which the medium was replaced with DMEM/high glucose supplemented with 10% FBS and 1% PS.

### Cardiomyocytes treatment

Before any treatment, neonatal cardiomyocytes were cultured in the replaced medium for 24 h. NETs concentration was determined by Picogreen dsDNA assay kits (P7589, Invitrogen, USA) and adjusted to 5–10 μg/mL. Neonatal cardiomyocytes were incubated in the medium containing NETs for further analysis, whereas the same volume of normal culture medium was used as blank and DNase I degraded NETs as the negative control. Treatments were conducted for 24 hours.

### Isolation of rat neutrophils from bone marrow

The femur and tibia of rats (2-3) were detached from the other connected tissues and washed three times in Hank’s balanced salt solution (HBSS). A Syringe was then used to rinse the bone marrow with Roswell Park Memorial Institute (RPMI) medium. Lysis of red blood cells (RBCs) eliminated the RBC contamination. Percoll gradients of 55%, 65%, 70%, and 80% were used to isolate neutrophils. The 70% Percoll fraction, including the cell layers at the boundaries, was collected and washed with HBSS for further use.

### Acquisition of rat NETs

A total of 0.5 × 10^8^–1 × 10^8^ isolated neutrophils were resuspended in 4 mL RMPI (supplemented with 10% FBS and 1% PS) in 10 cm plates and incubated with 500 nM PMA (P1585, Sigma, USA) for 3 hours to activate NETosis. For the negative control, DNase I (10 U/mL; 10104159001, Roche, USA) was used to degrade NETs. To collect NETs, the medium was removed, and NETs attached to the plate were washed gently with HBSS once, followed by intense flushing with fresh medium to detach the NETs from the plate. The washing medium was then collected and pipetted frequently for complete resuspension. Floating cells were removed by centrifuge at 300 g for 10 min. Finally, the suspension containing NETs was stored at −20 °C and used within 2 weeks.

### Isolation, culture and treatments of human neutrophils

To isolate human neutrophils, the isolation strategy was based on Polymorphrep (1114683, Axis-Shield, Norway). Peripheral blood (5 ml) was added onto the same volume of Polymorphrep in 15 mL centrifuge tubes and centrifuged at 800 g × 30 min at room temperature. Neutrophils between the Polymorphrep solution and water were collected and the remaining RBCs were lysed. Neutrophils were resuspended in RPMI + 10% FBS + 1% PS for immediate use. As the positive control, neutrophils were routinely incubated with 100 nM PMA. However, when analyzing the synergistically stimulating effects of Ang II on NETosis, only 10 nM PMA was used. For Ang II incubation, concentrations varied from 0.1 to 100 μM.

### Live cell 3D holotomographic microscopy

For live cell 3D holotomography, cardiomyocytes were settled onto a 35 mm plate with glass bottom (diameter = 15 mm). The culture medium was discarded, cells were washed three times with HBSS, and the medium containing NETs was then added. The acquisition was set for refractive index (RI; 3D tomography) for a time-lapse of 15 h every 15 min in a Nanolive Fluo-3D Cell Explorer® (Nanolive, Switzerland) microscope. At the end of the experiment, images were exported using Steve software v.2.6® (Nanolive). For zoomed video, the region of interest was cropped and the same procedure described above was applied.

### Celigo cytometry

The Celigo Image Cytometer instrument has been used in high-throughput cell-based assays.^52,53^ In brief, a transmission and epifluorescence optical setup for one bright-field (BF) and four fluorescence (FL) imaging channels (blue, green, red, and far red) were included and optimized for analysis. The fluorescence filter was set for the corresponding colors as follows: blue (EX: 377/50 nm, EM: 470/22 nm), green (EX: 483/32 nm, EM: 536/40 nm), red (EX: 531/40 nm, EM: 629/53 nm), and far red (EX: 628/40 nm, EM: 688/31 nm). Auto and manual focus were provided, with the focus found in BF. After the focus was registered, highly uniform images of the entire plates (including 6, 12, 24, and 96-well plates) were captured rapidly at different channels. For analysis of NETs formation, Hoechst (33342, ThermoFisher, USA) and SYTOX Green (S7020, Thermofishe) were added to reach final concentrations of 10 ng/mL and 300 nM, and incubated for 30 and 10 min, respectively.^54^ For the analysis of cell death, besides Hoechst Propidium Iodide (PI, KGA107, component 2; Annexin V-FITC/PI apoptosis detection kits, KeyGEN Bio TECH, China) was diluted 1:100 and added just before analysis according to manufacturer’s instruction. For analysis of mitochondrial ROS, cells were incubated with Hoechst and 10 μM MitoSOX (M34152, ThermoFisher) for 30 min. The fluorescent dyes were mixed by gentle pipetting.

### Transmission electron microscope

Samples were fixed with three percent glutaraldehyde for 24 hours, followed by postfixed with one percent osmium tetroxide. Samples were subsequently dehydrated in series acetone and infiltrated in Epon 812 for 30 to 60 minutes each time, which were finally embedded. The semithin sections were stained with methylene blue and ultrathin sections were cut with a diamond knife, and stained with uranyl acetate and lead citrate. Sections were examined with JEM-1400-FLASH Transmission Electron Microscope.

### Measurement of mitochondrial membrane potential

The membrane potential of mitochondria was determined with the JC-1 probe (KGA604, Mitochondrial membrane potential detection kits, KeyGEN) as previously described. Briefly, cells were incubated with 500 nM JC-1 according to the manufacturer’s recommendation. Images were observed through fluorescence microscopy and photos were obtained (Olympus, Japan). The intensity ratio of green fluorescence vs red fluorescence was calculated using the Image J software (Image J1, National Institutes of Health). And the fluorescence intensity was also detected by the fluorescence microplate reader (BMG Labtech, Germany).

### Western-blot analysis

Tissue homogenates and cell lysates were subjected to western blot to screen for fibrosis and NETs formation. Target proteins were separated from the lysates, transferred to a PVDF film and determined through chemiluminescence. The antibodies used included the following: anti-GAPDH (60004, Proteintech, China), anti-Collagen I (ab34710, Abcam), anti-p-p38 (4511 S, CST), anti-p38 (8690 S, CST), anti-p-Smad2 (ab188334, abcam), anti-Smad2 (ab33875, abcam), anti-p-Smad3 (9520 S, CST), anti-Smad3 (ab40854, Abcam), anti-Caspase 3 (9662 S, CST), anti-Cleaved-caspase 3 (9661 S, CST), anti-Beclin-1 (3495 S, CST), anti-p62 (ab56416, Abcam), anti-LC3B (2775 S, CST), anti-Cit-H3 (ab5103, Abcam), and anti-H3 (4499 S, CST) antibodies.

### Tachypacing of rat neonatal cardiomyocytes

Rat neonatal cardiomyocytes assigned to the normal control group were subjected to 1 Hz (normal pacing), 40 V and 10-ms pulses. For cardiomyocytes of the tachypacing group, the pacing rate was increased to 6 Hz, and the other parameters were unchanged. The C-pace EP culture stimulator was used to fire programmed pacing signals and the duration was usually less than 6 hours.

### mtDNA analysis

The supernatants (100 mL) were mixed with PBS (100 mL), followed by brief vortexing. The mixture was centrifuged at 700 *g* for 5 min at 4 °C, and the supernatants were carefully collected by avoiding touching the pellets with pipette tips. Lastly, the supernatants were further centrifuged at 18,000 *g* at 4 °C for 15 min, The resulting supernatants were further processed for DNA isolation using the DNeasy blood and tissue kits (#69504, Qiagen, USA) according to the manufacturer’s instruction. To determine the mtDNA in cell-free DNA, quantitative real-time polymerase chain reaction (qPCR) was then performed. Primer (mtDNA, *nadh dhe-1, nd-1*): forward TGGCCTTCCTCACCCTAGTA; reverse TTAGGGGGCGTATGGGTTCT. The plasma mtDNA concentration was:

$${\rm{c}}={\rm{Q}}* \frac{{Vdna}}{{Vpcr}}* \frac{1}{{Vext}}$$

Q is the number of determined mitochondrial DNA copies, V_dna_ is the total volume of plasma DNA obtained after extraction, V_pcr_ is the volume of plasma DNA solution used for PCR, and V_ext_ is the volume of plasma extracted. To compare the relative concentrations, we first calculated the average concentration of the normal control and then calculated the relative concentration ratios for comparison.

### Rat model of AF induced by Ach-CaCl_2_

SD rats were randomly assigned to the blank, AF or AF + DNase I groups. The pharmacological induction of AF in rats was described previously.^55–57^ Briefly, the AF group was induced with Ach (66 μg/mL)-CaCl_2_ (50 mg/mL) with 0.1 mL/100 g dose via the tail vein injection for 28 days. In the first Four days, rats were selected for the successful establishment of the AF model and rats without AF were excluded. The remaining rats were randomly assigned to group AF or AF + DNase I. The AF + DNase I group was administrated DNase I by intravenous injection once a day for the following 24 days at a therapeutic dose (0.1 mg/kg); an hour later, Ach-CaCl_2_ was re-administered.^58^ Electrocardiogram showed that the P wave disappeared and the f wave appeared as in the typical AF ECG. The P wave appeared and the f wave disappeared as a sign of AF to restore sinus rhythm. For sample collection, rats were anesthetized, peripheral blood was drained from the right ventricle and left atrium tissues were collected.

### Statistical analysis

Results are presented as the mean ± standard deviation. Groups were compared through analysis of variance and the Student’s t-test. All statistical analyses were performed using Prism 7 software (GraphPad Software, La Jolla, CA, USA). *P* < 0.05 was considered statistically significant. Data are presented as mean ± SD.

## Supplementary information

Supplementary information

supplementary movie 1

supplementary movie 2

## Data Availability

All data generated or analyzed during this study are included in the article.
