# Supplementary material for: Interaction between neutrophil extracellular traps and cardiomyocytes contributes to atrial fibrillation progression
Source: Signal Transduct Target Ther. 2023 Jul 26;8:279. doi: 10.1038/s41392-023-01497-2 (PMC10368710; doi:10.1038/s41392-023-01497-2)
Supplement: Supplementary file 1 — Supplementary information [file 41392_2023_1497_MOESM1_ESM.docx]

Supplementary Materials for

Interaction between Neutrophil Extracellular Traps and Cardiomyocytes Contributes to Atrial Fibrillation Progression

Li He†, Ruiqi Liu†, Honghua Yue, Xiaoxin Zhang, Xiaohui Pan, Yutao Sun, Jun Shi, Guonian Zhu, Chaoyi Qin*, Yingqiang Guo*

Correspondence to: qinchaoyi@wchscu.cn, drguoyq@wchscu.cn

**This PDF file includes:**

Supplementary Figures 1 to 10

Captions for Supplementary Movies 1 to 2

Supplementary Figure 1. | **CCK-8 analysis of cardiomyocytes treated with NETs, NETs+ mito-TEMPO and NETs+3-MA.** CCK-8 analysis showing a contrary trend of PI staining indicated NETs could increase NAD+ level and ROS production. Statistical significance was determined by unpaired two-tailed t-test. Experiment was repeated independently at least 3 times. Source data are provided as a Source Data file. n=5, ***P*﹤0.01, ****P*﹤0.001, **** *P*﹤0.0001, ns (not significant). Data were presented as mean ± SD

**
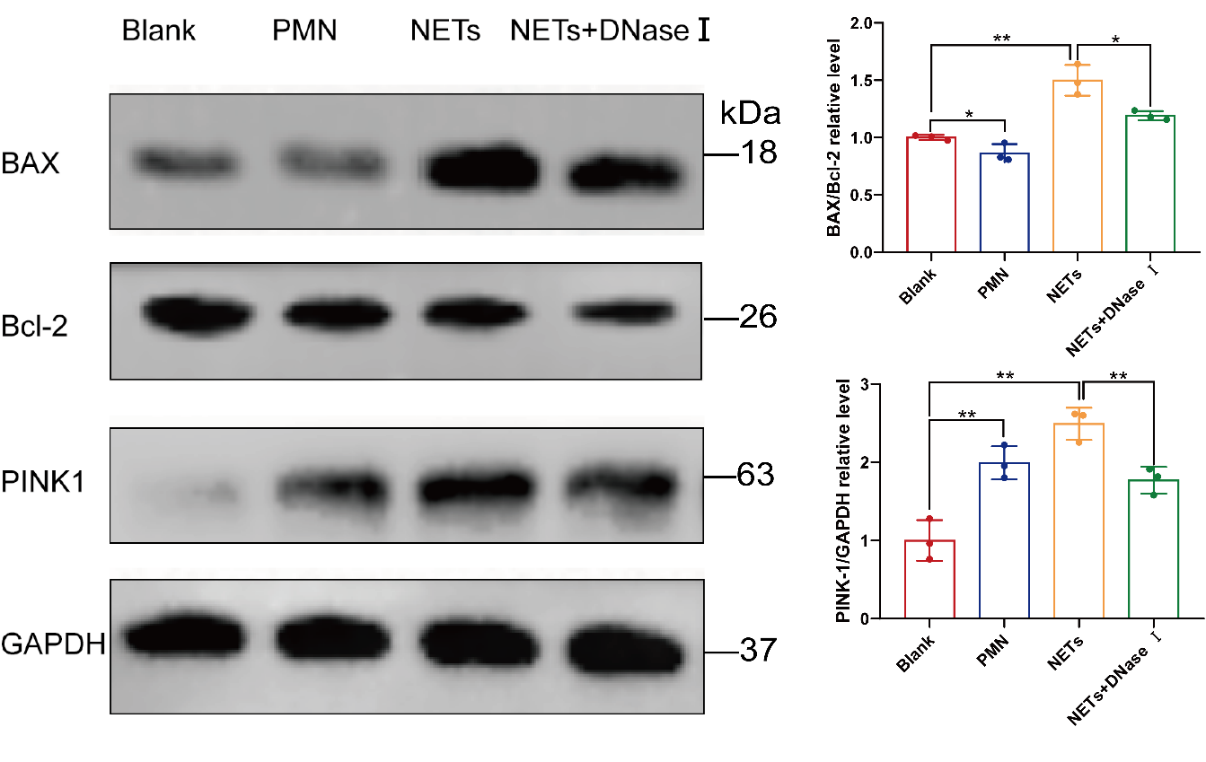
**

Supplementary Figure 2. | **NETs induced mitochondrial injury of cardiomyocytes.** *In vitro* NETs upregulate BAX/Bcl-2 and PINK1 in cardiomyocytes analyzed through WB. Statistical significance was determined by unpaired two-tailed t-test. Source data are provided as a Source Data file. n=3, **P*﹥0.05, ***P*﹤0.01. Data are presented as mean ± SD.

Supplementary Figure 3. | **Death rate of cardiomyocytes in the presence and absence of NETs and mito-TEMPO measured by Celigo analysis.** Cells cultured in 96-well plate were stained for all DNA (blue) and nucleuses of dead cells (red). Hoechst single positive cell was defined as the living, while Hoechst and PI double positive cell was defined as the dead. Statistical significance was determined by unpaired two-tailed t-test. Experiment was repeated independently at least 3 times. Source data are provided as a Source Data file. n=4, **P*﹤0.05, ***P*﹤0.01. Data are presented as mean ± SD.

Supplementary Figure 4. | **Low concentration of Ang Ⅱ (from 0.01 nM to 1 nM) could not induce significant increase of cfDNA secreted from neutrophils.** Neutrophils deprived from peripheral blood of donator cultured in 96-well plate was stained for all DNA (blue) and cfDNA (green). Hoechst single positive cell was defined as the living, while Hoechst and SYTOX Green double positive with linear DNA structure formation was defined as NETs secreted. Statistical significance was determined by unpaired two-tailed t-test. Experiment was repeated independently at least 3 times. Source data are provided as a Source Data file. n=4, ****P*﹤0.001, ns (not significant). Data are presented as mean ± SD.


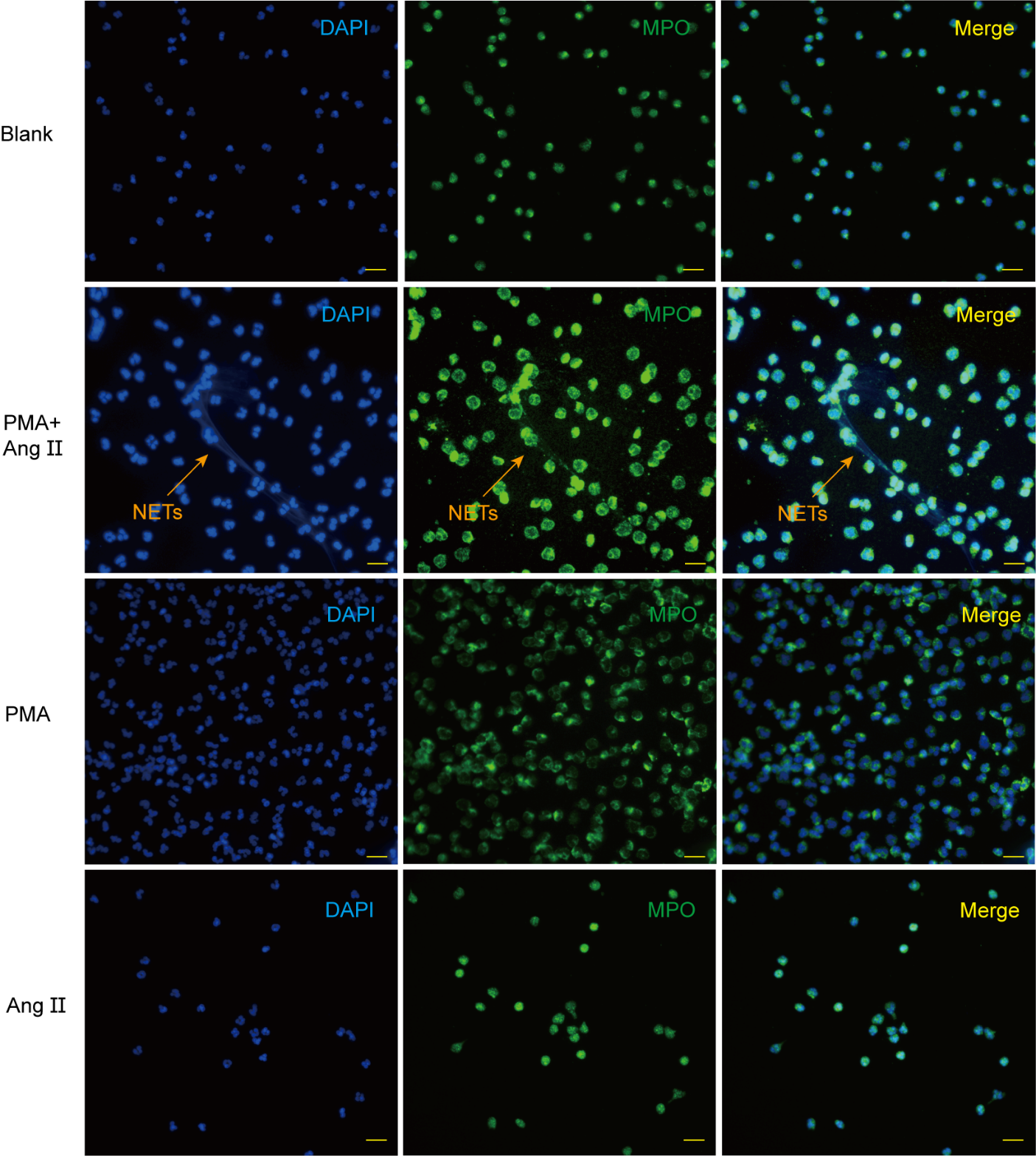


Supplementary Figure 5. | **Ang Ⅱ promotes NETosis.** Analysis of effect of Ang Ⅱ on NETosis induced by PMA determined by IF staining microscopy. Cells in 24-well plate on coverslips were stained for DNA (blue) and MPO (green). Yellow arrows indicate NETs. Scale bar: 20 μm.

Supplementary Figure 6. | **NETs secretion induced by supernatants of tachy-paced cardiomyocytes could be more potent than PMA.** Neutrophils deprived from rat bone marrow cultured in 96-well plate in the presence or absence of PMA, Ang Ⅱ and supernatants of cardiomyocytes paced with frequency of 1 Hz or 6 Hz was stained for all DNA (blue) and cfDNA (green). Hoechst single positive cell was defined as the living, while Hoechst and SYTOX Green double positive with linear DNA structure formation was defined as NETs secreted. Statistical significance was determined by unpaired two-tailed t-test. Experiment was repeated independently at least 3 times. Source data are provided as a Source Data file. n=3, ***P*﹤0.01, **** *P*﹤0.0001. Data are presented as mean ± SD.


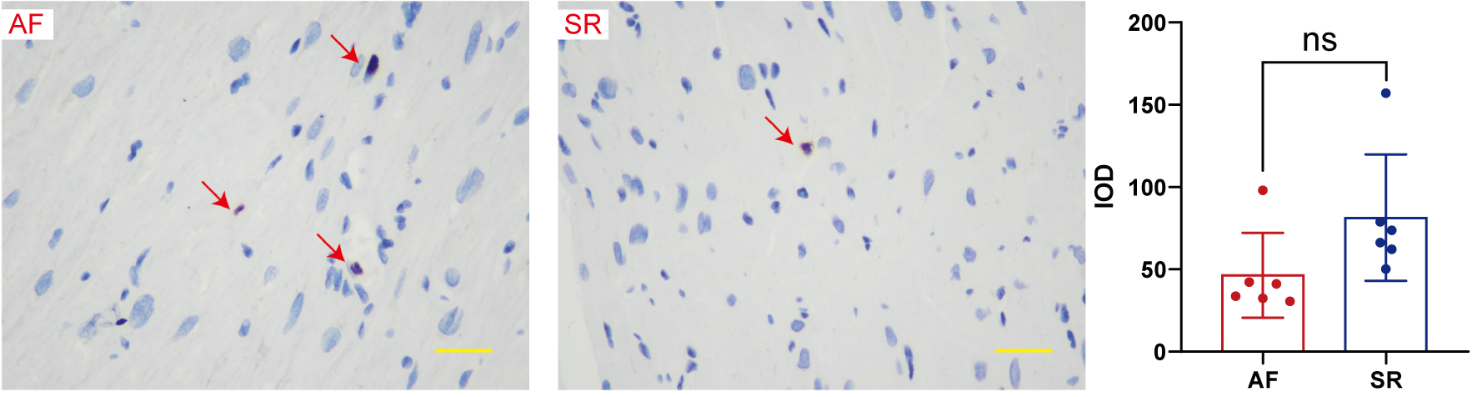


Supplementary Figure 7. | **Infiltrated neutrophils in atrial appendages in SR and AF patients.** Representative immunohistochemical staining of left atrial appendage for AF and SR group, Magnification ×400. The integral optical density of AF and SR sections: The infiltrating neutrophils were evaluated by the IOD of sections of AF and SR. There is no significant difference between the two groups. n=6, ns (not significant). Data are presented as mean ± SD.

**
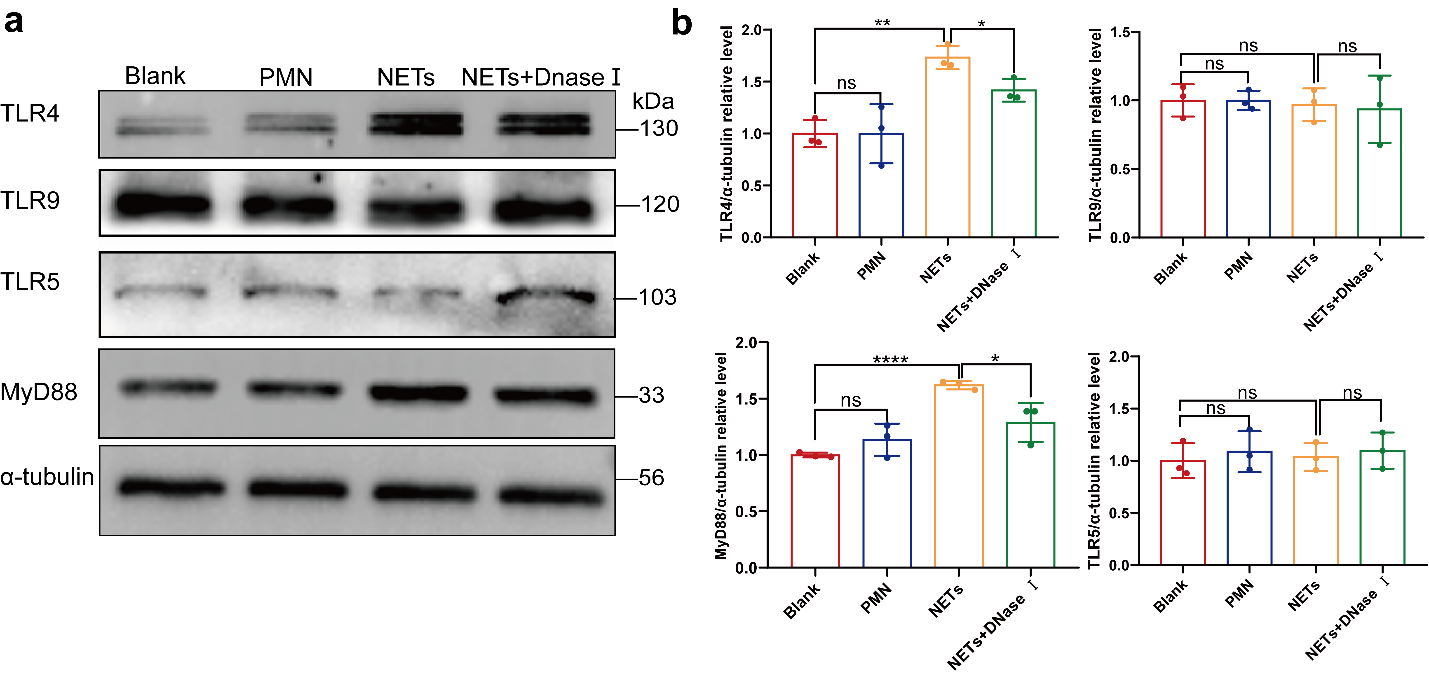
**

Supplementary Figure 8. | **TLR4/MyD88 signaling pathway in cardiomyocytes activated by NETs. a, b** Analysis of TLR4, TLR9, TLR5 and MyD88 of cardiomyocytes by Western Blot. The Western Blot shown is representative of three independent biological replicates. Cardiomyocytes were cultured in 6-well plate in the presence or absence of NETs, degraded NETs with DNase Ⅰ and supernatants of PMN. Statistical significance was determined by unpaired two-tailed t-test. Source data are provided as a Source Data file. n=3, **P*﹥0.05, ***P*﹤0.01, **** *P*﹤0.0001, ns (not significant). Data are presented as mean ± SD.


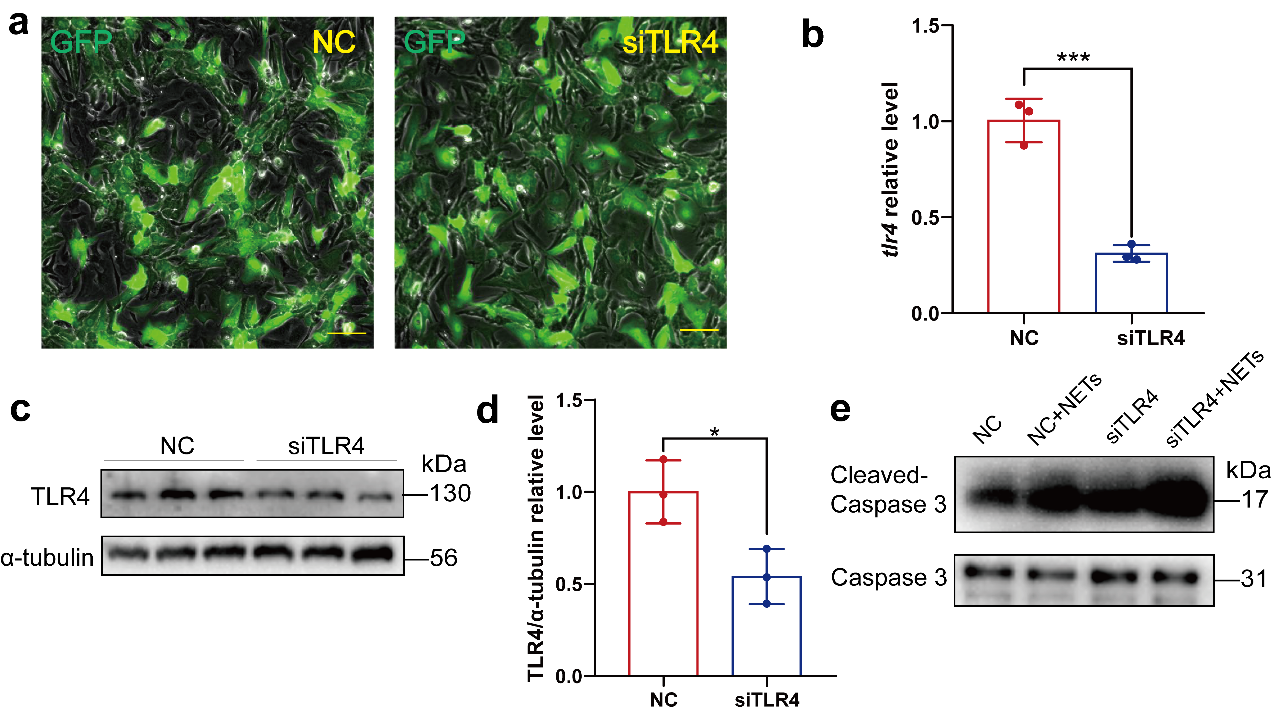


Supplementary Figure 9. | **TLR4 protects cardiomyocytes from NETs-induced apoptosis.** a-d TLR4 Knockdown in rat neonatal cardiomyocytes with siRNA. Rat neonatal cardiomyocytes cultured in 6-well plate was transfected with shRNA knocking down TLR4 carried by AV. The success of TLR4 knockdown was verified by IF microscopy, qPCR and Western Blot. Scale bar: 40 μm. e TLR4 knock-down further upregulated caspase 3 cleavage induced by NETs in cardiomyocytes. The Western Blot shown is representative of three independent biological replicates. Statistical significance was determined by unpaired two-tailed t-test. Source data are provided as a Source Data file. n=3, * *P*﹤0.05. Data are presented as mean ± SD.

**
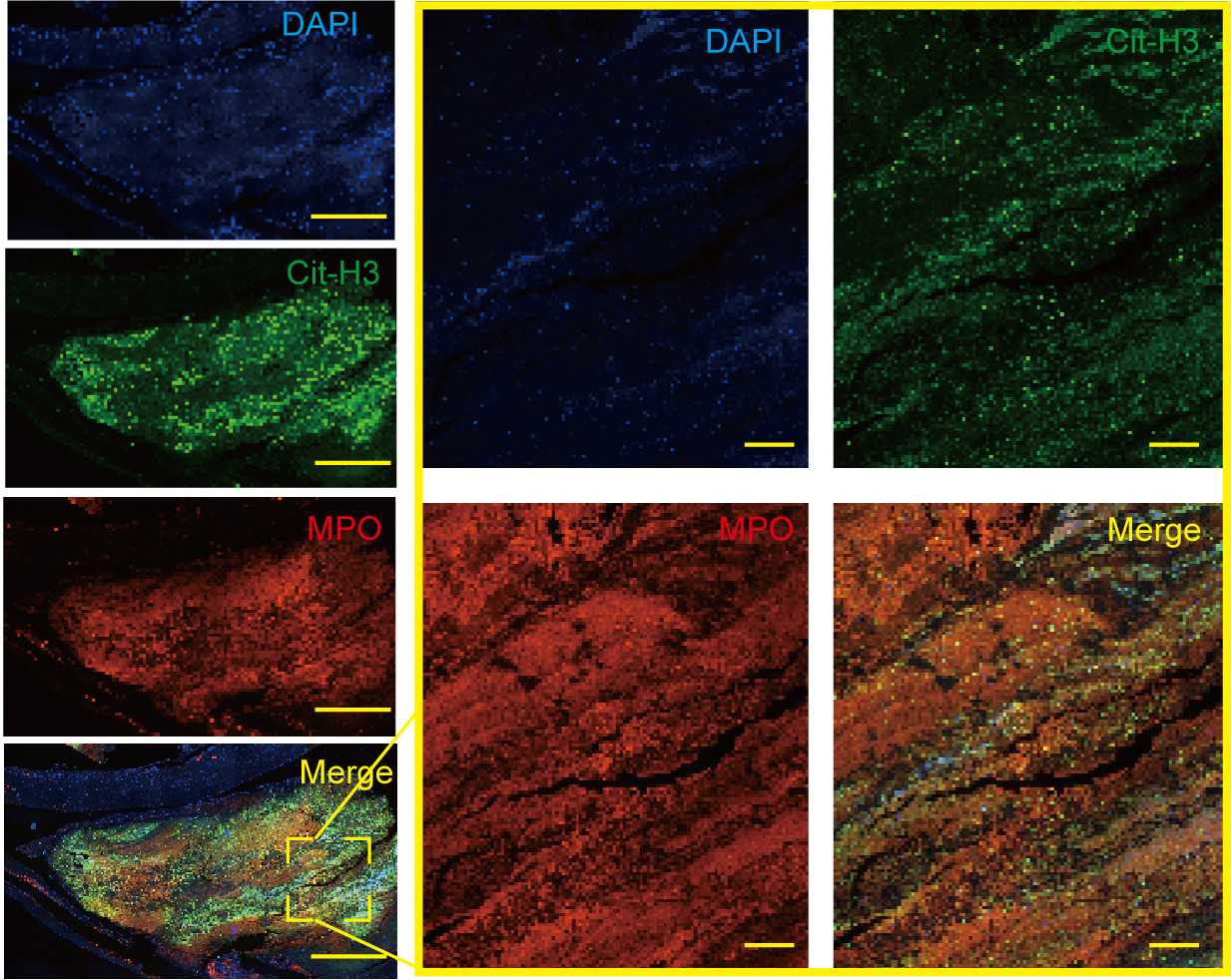
**

Supplementary Figure 10. | **NETs participate in the formation of mural thrombus in LA of AF patients.** NETs mainly located in the middle layer of mural thrombus. Paraffin section of mural thrombus obtained from LA of patients with AF was stained for DNA (blue), cit-H3 (green) and MPO (red). Linear structure with cit-H3 and MPO double positive was identified as NETs. Scale bar: 2 mm (left) or 200 μm (right). Source data are provided as a Source Data file.

Supplementary Movie 1.

**NETs induce atrophy of cardiomyocytes and increase of perinuclear granules.** Cardiomyocytes cultured in 35mm plate with glass bottom incubated with NETs was continually imaged by Nanolive Fluo-3D Cell Explorer® (Nanolive) microscope.

Supplementary Movie 2.

**Cardiomyocytes undergoing normal pacing and tachy-pacing.** Rat neonatal cardiomyocytes culture in 6-well plate were paced by C-Pace EP Culture Stimulator of 1 Hz (NC) or 6 Hz (TP).
